# Frequency and Circadian Timing of Eating May Influence Biomarkers of Inflammation and Insulin Resistance Associated with Breast Cancer Risk

**DOI:** 10.1371/journal.pone.0136240

**Published:** 2015-08-25

**Authors:** Catherine R. Marinac, Dorothy D. Sears, Loki Natarajan, Linda C. Gallo, Caitlin I. Breen, Ruth E. Patterson

**Affiliations:** 1 Moores UCSD Cancer Center, University of California San Diego, La Jolla, California, United States of America; 2 Graduate School of Public Health, San Diego State University, San Diego, California, United States of America; 3 Department of Family Medicine & Public Health, University of California San Diego, La Jolla, California, United States of America; 4 Department of Medicine, Division of Endocrinology and Metabolism, University of California San Diego, La Jolla, California, United States of America; 5 Department of Psychology, San Diego State University, San Diego, California, United States of America; The University of Manchester, UNITED KINGDOM

## Abstract

Emerging evidence suggests that there is interplay between the frequency and circadian timing of eating and metabolic health. We examined the associations of eating frequency and timing with metabolic and inflammatory biomarkers putatively associated with breast cancer risk in women participating in the National Health and Nutrition Examination 2009–2010 Survey. Eating frequency and timing variables were calculated from 24-hour food records and included (1) proportion of calories consumed in the evening (5pm-midnight), (2) number of eating episodes per day, and (3) nighttime fasting duration. Linear regression models examined each eating frequency and timing exposure variable with C-reactive protein (CRP) concentrations and the Homeostatic Model Assessment of Insulin Resistance (HOMA-IR). Each 10 percent increase in the proportion of calories consumed in the evening was associated with a 3 percent increase in CRP. Conversely, eating one additional meal or snack per day was associated with an 8 percent reduction in CRP. There was a significant interaction between proportion of calories consumed in the evening and fasting duration with CRP (p = 0.02). A longer nighttime fasting duration was associated with an 8 percent lower CRP only among women who ate less than 30% of their total daily calories in the evening (*p* = 0.01). None of the eating frequency and timing variables were significantly associated with HOMA-IR. These findings suggest that eating more frequently, reducing evening energy intake, and fasting for longer nightly intervals may lower systemic inflammation and subsequently reduce breast cancer risk. Randomized trials are needed to validate these associations.

## Introduction

There is compelling evidence that persistent low-grade inflammation is a significant underlying contributor to numerous cancers [1–4] (including breast) and is associated with several chronic metabolic conditions linked to breast cancer (e.g., obesity, diabetes) [5, 6]. Laboratory studies have demonstrated that chronic inflammation predisposes tissue to cancer development, whereby tumors generally arise within inflamed tissues [7]. Consistent with this evidence, data from epidemiologic cohorts of women have shown that elevated C-reactive protein (CRP), a biomarker of systemic inflammation, is associated with breast cancer risk [3, 4]. For example, a case-control study among postmenopausal women enrolled in the Multiethnic Cohort Study (706 cases; 706 controls) found that women in the highest quartile of CRP were significantly more likely to develop breast cancer than women in the lowest quartile (OR: 1.41; 95% CI 1.01–1.96; P_trend_ = .01) [4].

Insulin resistance is another metabolic condition that appears to play a significant role in the etiology of breast cancer and related metabolic diseases. Although the exact mechanisms linking insulin resistance to these conditions are unknown, experimental data indicate that elevated circulating insulin within the hyperinsulinemic range is a fundamental component of these links [8, 9]. For example, in a sample of women enrolled in the Women’s Health Initiative Observational Study, investigators found that women in the highest tertile of baseline insulin had nearly double the risk of breast cancer relative to women in the lowest tertile (HR: 2.22; 95% CI 1.39–3.53) [9]. The authors of this study also reported that the estimate of breast cancer risk based on insulin was even higher in the subsample of women who did not use hormone replacement therapy (HR: 3.15; 95% CI 1.61–6.17). Insulin resistance is also a hallmark of type 2 diabetes and type 2 diabetes risk [10], and has been associated with numerous other cancers such as liver, kidney, and pancreatic cancer [11].

Recent evidence suggests that dietary behaviors such as eating frequency and timing influence insulin secretion and systemic inflammation, may have downstream effects inflammation and insulin resistance. The majority of this initial evidence has come from mechanistic studies in animals. Landmark studies in rodents have demonstrated that eating regimens that restrict food intake to within a 4- to 8-hour window of time (i.e., ‘time-restricted feeding’), thereby increasing the number of hours spent fasting, can improve metabolic processes related to insulin resistance and inflammation. Specifically, mice subjected to these feeding-fasting regimens appear to be protected from hyperinsulinemia, hepatic steatosis, and inflammation [12]. Notably, the most profound effects of these feeding-fasting regimens on metabolism seem to occur when synchronized with daily sleep-wake cycles (i.e., eating only during the night and fasting during the day for nocturnal mice) [13]. To date, only small-scale human studies have examined the impact of fasting regimens on human metabolic health. Results of these studies generally indicate that fasting has favorable effects on insulin resistance parameters but provide little data on inflammation [14]. It is difficult to extrapolate from these small-scale studies of fasting to our research question regarding the health impacts of prolonged nightly fasting. However, our recently published analysis of NHANES data did find that prolonged nightly fasting was associated with significantly lower levels of HbA1c [15].

Although data from human studies are sparse, there is evidence that other eating frequency and timing behaviors can influence biomarkers of breast cancer and metabolic disease risk. In particular, eating large meals in the evening appears to have deleterious effects on insulin metabolism [16, 17]. A cross-over study among 6 healthy men found insulin sensitivity to be significantly impaired among men consuming a high-energy (60 percent of daily calories) meal at dinner vs. in the morning [17]. A 12-week randomized parallel-arm trial among 93 overweight and obese women found that insulin (fasting and postprandial) and HOMA-IR were significantly improved when the women ate a high-calorie breakfast vs. high-calorie dinner (total daily caloric intake was the same) [16]. Evidence from controlled feeding studies generally suggests that grazing (small, frequent meals) as opposed gorging (fewer, large meals) may improve regulation of metabolic processes [18, 19]. For example, a recent trial among 54 patients with type 2 diabetes randomized to isocaloric diets consumed as either 2 meals or 6 meals per day found that more frequent meals resulted in significantly reduced body weight and fasting glucose concentrations [20]. Less is known about optimal meal frequency in nondiabetics, and recommendations on meal frequency for reducing chronic disease risk remain highly controversial.

The purpose of this study was to further examine the associations between eating frequency and timing behaviors with inflammation and insulin resistance biomarkers putatively associated with increased breast cancer risk. Eating frequency and timing variables were (1) proportion of calories consumed in the evening (between 5pm and midnight); (2) number of eating episodes per day; and (3) nighttime fasting duration (a time-restricted feeding schedule that is aligned with sleep-wake cycles). We used data from a population-based sample of women participating in the 2009–2010 National Health and Nutrition Examination Survey.

## Materials and Methods

### Study Sample

Data for this study were obtained from the 2009–2010 National Health and Nutrition Examination Survey (NHANES), which is a continuous annual survey conducted by the National Center for Health Statistics. NHANES comprises a nationally representative sample of the U.S. civilian non-institutionalized population, selected by a complex, multistage, stratified, clustered probability design. The survey consists of two components: (1) an in-home interview; and (2) an in-person comprehensive medical examination at the mobile exam center, which includes an array of laboratory tests. The National Center for Health Statistics Research Ethics Review Board approval was granted and documented consent was obtained from all study participants. Details of the study procedures have been published elsewhere (http://www.cdc.gov/nchs/nhanes.htm) [21].

The current study sample consisted of 2,650 adult women from the NHANES 2009–2010 survey year who completed the in-person comprehensive medical exam at the mobile exam center. We excluded women who did not have the telephone-based dietary recalls in the online database, women who had diabetes or borderline-diabetes (self-report), were taking medication for diabetes, and women who were pregnant (n = 438 excluded). Outcomes requiring a fasting blood draw, such as fasting insulin and glucose (used to calculate HOMA-IR), were only available for a subsample of adult women who were scheduled for morning blood draws (HOMA-IR subsample; n = 1,034).

### Dietary Assessment

Eating behavior data used in our analyses were collected from each participant via a 24-hour dietary recall that was conducted by telephone 3–10 days after the in-person medical exam. The dietary recall was conducted in partnership between the U.S. Department of Agriculture (USDA) and the U.S. Department of Health and Human Services (DHHS). USDA’s Food Surveys Research Group (FSRG) was responsible for the dietary data collection methodology and database maintenance.

### Outcome and Covariate Assessment

#### C-reactive protein (CRP)

All blood specimens were obtained by trained medical professionals in mobile examination centers and were analyzed at the Fairview Medical Center Laboratory at the University of Washington. Participants were not required to fast prior to the collection of blood specimens used in the determination of CRP, given that this biomarker can be measured accurately in non-fasting conditions [22]. There were also no restrictions as to the time of day in which these blood specimens were collected, as CRP does not exhibit diurnal variation [23]. High-sensitivity assays performed on a latex-enhanced Behring Nephelometer were used to quantify CRP concentrations. CRP concentrations were calculated using a calibration curve. Data reduction of the signals was performed by using a storable logit-log function for the calibration curve.

#### HOMA-IR

Individuals who were assigned a morning examination provided fasting blood specimens for insulin and glucose measures. Blood specimens were collected after a 9-hour fast using standard procedures and were sent to the University of Minnesota for analysis. Insulin concentration was quantified using a Merocodia Insulin ELISA, which is a two-site enzyme immunoassay. Plasma Glucose concentrations measured by a hexokinase-mediated reaction Roche/Hirachi Modular P Chemistry Analyzer using plasma blood specimens. The Homeostatic Model Assessment of Insulin Resistance (HOMA-IR) was used to estimate the degree of insulin resistance in each participant using the following equation [24]:

HOMA−IR=fasting glucose (mml/L) × fasting insulin (uU/ml)/22.5

#### Eating frequency and timing

The number of eating episodes per day variable was defined as the number of time-stamps associated with calorie-containing food or beverage consumption. It is unclear how many ingested calories are needed for initiation of metabolic processes; therefore, we used a conservative 5 kcal cutoff to define an eating episode. We re-processed the dietary data using more liberal calorie cutoffs to define an eating episode (e.g., 10 kcal, 25 kcal); however these liberal cutoffs did not meaningfully change the study findings or conclusions rendered. Evening caloric intake was calculated by summing the total calories consumed between 5 pm and midnight. Nighttime fasting duration was estimated by (1) calculating the elapsed time between the first and last calorie-containing food or beverage consumed for each 24-hour dietary recall day; and (2) subtracting this elapsed time from 24.

#### Other covariates

The Family and Sample Person Demographics questionnaire ascertained demographic data on survey participants. This questionnaire was administered in the home, by trained interviewers using the Computer-Assisted Personal Interviewing system. Demographic covariates used in regression analyses include age (continuous variable), ethnicity (categorical variable: non-Hispanic white, non-Hispanic black, Mexican American, other Hispanic, and others), and education (did not complete high school, completed high school, and attended/completed college or advanced degree. Physical activity was assessed using the physical activity questionnaire, which includes questions related to daily activities, leisure time activities, and sedentary activities [25]. Responses were used to calculate an estimate of weekly metabolic equivalents (MET) using the analytic notes and suggested MET scores outlined in the NHANES online documentation (http://www.cdc.gov/nchs/nhanes.htm). Briefly, work-related activities and vigorous leisure-time physical activities were assigned MET values of 8.0; moderate work-related activities, walking or bicycling for transportation, and moderate leisure-time physical activities were assigned MET values of 4.0. Sleep duration was assessed using the single item question, “How much sleep do you usually get per night on weekdays or workdays?”). Height and weight measurements were obtained using standardized techniques and equipment during the medical exam.

### Statistical Analysis

Descriptive statistics were used to characterize the study population. Outcome variables were assessed for linearity and normality. Both HOMA-IR and CRP were non-normally distributed and were log-transformed to better approximate Gaussian distribution. Multivariable linear regression models were used to examine the joint-effects of the eating frequency and timing exposure variables (all three exposures modeled together) with the inflammation and insulin resistance biomarkers of interest (CRP and HOMA-IR). Models controlled for the primary confounders of age, education, and race/ethnicity.

We hypothesized that fasting is more beneficial when nighttime eating is restricted (i.e., fasting during the night and eating during the day). Therefore, we tested for effect modification between hours of nighttime fasting proportion of calories consumed in the evening, using tests of statistical interaction, and in models stratified by the proportion of evening caloric intake (<30% vs. ≥30%) based on quartile distributions (bottom quartile vs. upper three). Interaction and stratified models controlled for the same covariates described above, as well as the other eating frequency and timing variables.

When modeling CRP, we excluded individuals with CRP concentrations greater than or equal to 15 mg/L, given that our goal was to model chronic inflammation (very high CRP may indicate acute inflammation); however sensitivity analyses revealed that excluding individuals with high CRP did not influence the associations we report. We explored adjustment for physical activity, total caloric intake, as well as other dietary exposures (e.g., total fat, carbs), however, these variables were not statistical confounders and, thus, were not included final models. To ease interpretation and clinical relevance of the parameter estimates for the Evening Calories variable, we used a 10 percent unit of analysis. Using this approach, a 10 percent increase in the proportion of total calories consumed in the evening (after 5 pm) corresponds to a 1-unit change in the biomarker outcome of interest. Data were analyzed using SAS Studio and SAS version 9.4 and (Carey, NC). Sample weights were used in all analyses to account for differential probabilities of selection into the sample, nonresponse, and noncoverage. Standard errors were estimated using Taylor Series Linearization. All statistical tests were set at an overall significance level cutoff of *p*<0.05.

## Results

Sociodemographic, behavioral, and biomarker characteristics of the eligible (n = 2,212), excluded (n = 438), and HOMA-IR subsample (n = 1,034) are presented in Table 1. The primary reason women were excluded from the analytic sample was due to having diabetes (self-report) and/or taking antidiabetic medication. The mean age of women in the eligible sample was 46.8 (SEM = 0.7) years. They consumed an average of 774 (SEM = 17.1) kcals between 5 pm and midnight, fasted 12.4 hours per night (SEM = 0.1), and ate 4.7 (SEM = 0.1) times per day. These characteristics for the HOMA-IR subsample were similar to those of all eligible women.

**Table 1:** Demographic, Lifestyle, and Dietary Characteristics of a Nationally-Representative Sample of Adult Women From the NHANES 2009–2010 Survey.

Characteristics mean (SEM) unless otherwise noted	Eligible n = 2,212	Excluded n = 438	HOMA-IR^a^ n = 1,034
**Age**	46.8 (0.7)	52.5 (0.9)	47.2 (0.7)
**Ethnicity** n(%)			
Non-Hispanic White	1,138 (51.4)	164 (38.1)	514 (49.7)
Non-Hispanic Black	355 (16.0)	106 (24.7)	151 (14.6)
Mexican American	379 (17.1)	93 (21.6)	195 (18.9)
Other race(s)	340 (15.4)	75 (17.4)	174 (16.8)
**Education** ^b^ n(%)			
No High School Diploma	549 (24.8)	161 (37.4)	252 (24.4)
High School Diploma	494 (22.3)	93 (21.6)	225 (21.8)
Some College	682 (30.8)	127 (29.5)	322 (31.1)
College Degree	483 (21.8)	54 (12.6)	233 (22.5)
**BMI**	28.2 (0.2)	33.4 (0.6)	28.4 (0.2)
**Sleep (hours)**	7.0 (0.0)	7.0 (0.1)	7.0 (0.1)
**Daily Energy Intake (kcal/day)**	1,773.1 (22.5)	1,636.6 (52.9)	1,772.5 (20.7)
**Daily Energy Intake After 5pm (kcal/day)**	773.7 (17.1)	677.9 (27.5)	772.0 (18.4)
**Nighttime Fasting Duration (hours)**	12.4 (0.1)	13.0 (0.2)	12.2 (0.1)
**Number of Eating Episodes per day**	4.7 (0.1)	4.4 (0.1)	4.7 (0.1)
**CRP** ^c^ **(median Q1,Q3)**	0.4 (0.6, 0.4)	0.7 (0.2, 0.8)	0.4 (0.1, 0.5)
**Insulin (median Q1,Q3)**	—	16.8 (9.3, 20.8)	12.1 (6.2, 15.1)
**Glucose (median Q1,Q3)**	—	6.8 (5.3, 7.7)	5.4 (4.9, 5.6)
**HOMA-IR** ^a^ **(median Q1,Q3)**	—	5.2 (2.4, 6.1)	3.0 (1.4, 3.7)

^a^Homeostatic Model Assessment of Insulin Resistance.

^b^Data on educational attainment was missing for 4 study participants.

^c^C-reactive protein.

Eating frequency and timing variables were generally modestly but statistically significantly correlated (data not shown). According to weighted Pearson correlation coefficients, eating frequency was positively correlated with the Evening Calories variable (r = 0.2, *p*< 0.001) and inversely associated with nighttime fasting duration (r = -0.51, *p*<0.001). Nighttime fasting duration was also inversely correlated with Evening Calories (r = -0.15, *p*<0.001). However, statistical tests revealed no evidence of collinearity (low variance inflation factor [<3]). There were also statistically significant positive associations between the CRP and HOMA-IR (r = 0.45, *p*<0.001), suggesting that the two biomarker outcomes are distinct yet moderately related.

Associations of eating frequency and timing exposure variables with CRP and HOMA-IR are presented in Table 2. As described previously, the parameter estimates for the Evening Calories variable should be interpreted as 1 point increase/decrease in the outcome of interest (CRP or HOMA-IR), per 10 percent increase in the proportion of total daily calories that were consumed in the evening (after 5 pm). The back-transformed parameter estimates indicate that each 10 percent increase in proportion of calories consumed in the evening was associated with a 3 percent increase in CRP (*β* 1.03; 95% CI 1.01–1.06; *p* = 0.02). Eating frequency was inversely related to CRP. Specifically, eating one additional meal per day was associated with an 8 percent reduction in CRP (*β* 0.92; 95% CI 0.86–0.99; *p* = 0.03). Nighttime fasting duration was not significantly associated with CRP; and none of the eating frequency or timing behaviors were associated with HOMA-IR.

**Table 2:** Models* of associations of Eating Patterns and Lifestyle Factors with C-Reactive Protein (CRP) and Homeostatic Model Assessment of Insulin Resistance (HOMA-IR) in Adult Women Participants From the NHANES 2009–2010 Survey Year.

	CRP^a^ (n = 2,019)	
	*β* (95%CI)	*p*-value
Evening Calories^b^	1.03 (1.01–1.06)	0.02
Eating Frequency	0.92 (0.86–0.99)	0.03
Nighttime Fasting Duration	1.01 (0.97–1.05)	0.70
	HOMA-IR^a^ (n = 1,034)	
	*β* (95%CI)	*p*-value
Evening Calories^b^	1.00 (0.98–1.02)	0.96
Eating Frequency	0.97 (0.92–1.03)	0.26
Nighttime Fasting Duration	1.00 (0.98–1.02)	0.98

*Models controlled for age, race/ethnicity, and education.

^a^Parameter estimates have been back transformed to reflect the percent change in each outcome (CRP and HOMA-IR) associated with a 1-unit increase in each dietary exposure variable.

^b^Calories consumed in the evening (between 5pm and midnight), divided by total energy intake.

There was a statistically significant interaction between nighttime fasting and the Evening Calories variable (p = 0.02). Accordingly, exploratory models examined the association of nighttime fasting stratified by portion of calories consumed in the evening (<30% calories after 5pm vs. ≥30% calories)(Table 3). Among women who ate fewer than 30% of their calories in the evening, nighttime fasting duration was inversely associated with CRP concentrations. Specifically, according to the back-transformed parameter estimates, each additional hour of fasting was associated with an 8 percent lower concentration of CRP (*β* 0.92; 95% CI 0.87–0.98; *p* = 0.01); however this association was not statistically significant when evening calories were greater than or equal to 30 percent. Stratified models revealed no significant associations between nighttime fasting duration and HOMA-IR.

**Table 3:** Stratified Models* of Associations of Nighttime Fasting Duration with CRP and HOMA-IR, by Percent of Calories Consumed in the Evening^a^.

	CRP^b^			
	**Evening Calories < 30% (n = 543)**		**Evening Calories ≥ 30% (n = 1476)**	
	β (95%CI)	*p*-value	β (95%CI)	*p*-value
Nighttime Fasting Duration	0.92 (0.87–0.98)	.01	1.04 (1.00–1.08)	0.07
	HOMA-IR^b^			
	**Evening Calories < 15% (n = 295)**		**Evening Calories < 30% (n = 739)**	
	β (95%CI)	*p*-value	β (95%CI)	*p*-value
Nighttime Fasting Duration	0.98 (0.91–1.05)	0.48	1.01 (0.99–1.04)	0.91

*Models controlled for age, race/ethnicity, and education; as well as evening calories and eating frequency.

^a^Calories consumed in the evening (between 5pm and midnight), divided by total energy intake.

^b^Parameter estimates have been back transformed to reflect the percent change in each outcome (CRP and HOMA-IR) associated with a 1-unit increase in each dietary exposure variable.

## Discussion

The degree to which timing and frequency of food intake influences metabolic health and chronic disease risk is an exciting new area of dietary research [14]. Data from longitudinal studies suggests that over time, adults are eating a greater proportion of their daily calories in the evening [26], although this shift in the distribution energy intake towards the evening hours may have deleterious effects on human health [27]. The current study found significant associations between evening caloric intake and circulating concentrations of biomarkers of generalized inflammation. Specifically, each 10 percent increase in the proportion of daily calories consumed after 5 pm was associated with a significantly higher concentration of CRP—a biomarker that has been associated with increased risk of breast cancer, as well as a variety of chronic conditions [28]. This association between evening caloric intake and CRP was independent of potential lifestyle confounders, as well as the other dietary variables hypothesized to influence metabolic heath (e.g., nighttime fasting duration and eating frequency).

Animal and human studies have examined the impact of meal frequency on metabolism with conflicting results. In particular, although there is a wealth of evidence from rodent models that meal skipping (a form of intermittent fasting) has favorable effects on insulin metabolism and inflammation [13], meal frequency studies in humans have not supported these associations [14]. It is notable that in most instances, human studies were done over a short period with small sample sizes, limiting the conclusions that can be drawn. For example, a study by Rashidi and colleagues compared the effect of a diet composed of 3 meals vs. 9 snacks per day in 15 healthy men. After three weeks on these diets, men on the snacking diet had reduced fasting insulin concentration (*p*<0.01) [29]. In contrast, an 8-week controlled feeding study compared the effect of repeatedly consuming 1 vs. 3 meals per day in a sample of 10 healthy adult men and women and found no difference in fasting plasma insulin concentration or proinflammatory cytokine expression with respect to meal frequency [30]. The results of the current study were similarly mixed: we found no associations between eating frequency or fasting variables and insulin resistance (HOMA-IR); however we did observe a significant protective effect of increased eating frequency on plasma CRP concentration, a measure of systemic inflammation. It is possible that the lack of association observed between eating frequency and insulin resistance may be due to measurement error in the biomarker concentrations used to construct the HOMA-IR variable (i.e., fasting insulin and glucose). Specifically, although HOMA-IR is one of the most frequently used methods of determining insulin resistance in large population-based studies, it is approximated from single fasting glucose and insulin measurements, which may have a large amount of day-to-day variability compared to the euglycemic-hyperinsulinemic clamp method (gold standard approach for measuring insulin resistance). This variability could have obscured significant relationships between eating behaviors and insulin resistance parameters.

Accumulating evidence from studies in rodents suggest that eating patterns that restrict the number of hours that food can be consumed on a given day and increase the number of hours spent fasting have favorable effects on a number of parameters related to metabolic health [12]. These rodent studies also highlight the importance of aligning fasting regimens with sleep-wake cycles [12, 31, 32]. In particular, fasting patterns that reduce or eliminate nighttime eating have been shown to protect against a number of disease risk factors, including hyperinsullinemia, hepatic steatosis, and inflammation [12]. Similar to the evidence from rodent studies, we found that longer nighttime fasting intervals were associated with significantly lower concentrations of CRP and non-significantly lower concentrations of insulin resistance biomarkers. However, these favorable effects of nighttime fasting on inflammation and insulin resistance biomarkers were only present among a subsample of women who initiated their fast before 6 pm. Although the size of this subsample was relatively small and models were exploratory, these findings suggest that lengthening the nighttime fasting intervals may only be an effective disease prevention strategy when the nighttime fast is initiated early in the evening. In other words, eating late at night but skipping breakfast (which could also result in a long fasting interval) may be a biologically different behavior.

The underlying associations between meal frequency and timing variables with inflammation may involve circadian rhythm parameters. Feeding is a powerful signal (or ‘zeitgeber’), influencing alignment of circadian rhythms in peripheral tissues. Data from animal models have shown the eating at the ‘wrong’ circadian time (e.g., late at night) can reset some peripheral clocks almost entirely, resulting in a phase shift and misalignment of daily rhythms [31–33] and increased expression of proinflammatory genes such as *TNF-α*, *IL6*, and *CXCL2* [12]. Gene knockout studies have confirmed links between circadian disruption and inflammation: removal of the core clock component protein cryptochrome has been shown to result in constitutive elevation of proinflammatory cytokines [34]. In humans, controlled simulations of forced circadian misalignment have also demonstrated evidence of a causal link between circadian misalignment and inflammation. For example, a study by Wright and colleagues investigated the effect of forced circadian disruption in healthy men and women using a 25-day laboratory entrainment protocol. The authors of this study reported that plasma concentrations of proinflammatory proteins TNF-α and CRP were significantly higher during the period of forced circadian misalignment [35].

Although there are numerous advantages to using 24-hour dietary recalls for measuring dietary patterns, the limitations are noteworthy. For example, 24-hour dietary recalls are prone to response biases [36]. We are not aware of any published studies, however, that have documented random error or bias in self-report of meal timing. Diets may also vary considerably from day to day, and thus our measure of nighttime fasting duration derived from a single 24-hour recall likely has considerable measurement error. Research has also demonstrated that CRP measurements are susceptible to this same type of measurement error (day-to-day within-subject variability [37], and CRP concentrations can be elevated acutely by other factors, such as the common cold. Nevertheless, these random measurement errors would attenuate associations between dietary exposures and outcomes [36]; thus, it is likely that the parameter estimates and odd ratios presented in this study are conservative.

Strengths of this study include the diverse, population-based sample of women with extensive information on potential confounding demographic, clinical, and behavioral variables. Findings are also strengthened by the large sample size with sufficient power to detect associations between dietary exposures and biomarker outcomes of interest.

In conclusion, results of this study suggest that reducing evening energy intake, eating more frequently, and fasting for longer nightly intervals (when fasting is initiated early in the evening) may reduce systemic inflammation in the body which could subsequently reduce breast cancer and chronic disease risk. To our knowledge, this is the first population-based study to document these associations. Adequately powered randomized trials are needed to validate whether changing these meal frequency and timing behaviors significantly influence inflammatory processes. If the current findings are confirmed, reducing evening energy intake, eating more frequently, and fasting for longer nightly intervals (when initiated early in the evening), could be recommended as simple and effective behavioral strategies for breast cancer and disease prevention.

## References

[1] Coussens LM, Werb Z. Inflammation and cancer. Nature. 2002;420(6917):860–7. 10.1038/nature01322 12490959 PMC2803035

[2] Heikkila K, Ebrahim S, Lawlor DA. A systematic review of the association between circulating concentrations of C reactive protein and cancer. Journal of epidemiology and community health. 2007;61(9):824–33. 10.1136/jech.2006.051292 17699539 PMC2703800

[3] Dossus L, Jimenez-Corona A, Romieu I, Boutron-Ruault MC, Boutten A, Dupre T, et al C-reactive protein and postmenopausal breast cancer risk: results from the E3N cohort study. Cancer causes & control: CCC. 2014;25(4):533–9. 10.1007/s10552-014-0355-9 . 24504436

[4] Ollberding NJ, Kim Y, Shvetsov YB, Wilkens LR, Franke AA, Cooney RV, et al Prediagnostic leptin, adiponectin, C-reactive protein, and the risk of postmenopausal breast cancer. Cancer prevention research. 2013;6(3):188–95. 10.1158/1940-6207.CAPR-12-0374 23466816 PMC3595121

[5] Shoelson SE, Lee J, Goldfine AB. Inflammation and insulin resistance. The Journal of clinical investigation. 2006;116(7):1793–801. 10.1172/JCI29069 16823477 PMC1483173

[6] Patterson RE, Rock CL, Kerr J, Natarajan L, Marshall SJ, Pakiz B, et al Metabolism and breast cancer risk: frontiers in research and practice. Journal of the Academy of Nutrition and Dietetics. 2013;113(2):288–96. 10.1016/j.jand.2012.08.015 23127511 PMC3557584

[7] Shacter E, Weitzman SA. Chronic inflammation and cancer. Oncology. 2002;16(2):217–26, 29; discussion 30–2. . 11866137

[8] Gunter MJ, Hoover DR, Yu H, Wassertheil-Smoller S, Rohan TE, Manson JE, et al Insulin, insulin-like growth factor-I, and risk of breast cancer in postmenopausal women. Journal of the National Cancer Institute. 2009;101(1):48–60. 10.1093/jnci/djn415 19116382 PMC2639294

[9] Kabat GC, Kim M, Caan BJ, Chlebowski RT, Gunter MJ, Ho GY, et al Repeated measures of serum glucose and insulin in relation to postmenopausal breast cancer. International journal of cancer Journal international du cancer. 2009;125(11):2704–10. 10.1002/ijc.24609 . 19588485

[10] Kahn SE, Hull RL, Utzschneider KM. Mechanisms linking obesity to insulin resistance and type 2 diabetes. Nature. 2006;444(7121):840–6. 10.1038/nature05482 . 17167471

[11] Vigneri P, Frasca F, Sciacca L, Pandini G, Vigneri R. Diabetes and cancer. Endocrine-related cancer. 2009;16(4):1103–23. 10.1677/ERC-09-0087 . 19620249

[12] Hatori M, Vollmers C, Zarrinpar A, DiTacchio L, Bushong EA, Gill S, et al Time-restricted feeding without reducing caloric intake prevents metabolic diseases in mice fed a high-fat diet. Cell metabolism. 2012;15(6):848–60. Epub 2012/05/23. 10.1016/j.cmet.2012.04.019 22608008 PMC3491655

[13] Rothschild J, Hoddy KK, Jambazian P, Varady KA. Time-restricted feeding and risk of metabolic disease: a review of human and animal studies. Nutrition reviews. 2014;72(5):308–18. Epub 2014/04/18. 10.1111/nure.12104 . 24739093

[14] Patterson RE, Laughlin GA, Sears DD, LaCroix AZ, Marinac C, Gallo LC, et al Intermittent Fasting and Human Metabolic Health. Journal of the Academy of Nutrition and Dietetics. 2015 10.1016/j.jand.2015.02.018 . 25857868 PMC4516560

[15] Marinac CR, Natarajan L, Sears DD, Gallo LC, Hartman SJ, Arredondo E, et al Prolonged Nightly Fasting and Breast Cancer Risk: Findings from NHANES (2009–2010). Cancer epidemiology, biomarkers & prevention: a publication of the American Association for Cancer Research, cosponsored by the American Society of Preventive Oncology. 2015;24(5):783–9. Epub 2015/04/22. 10.1158/1055-9965.EPI-14-1292 25896523 PMC4417458

[16] Jakubowicz D, Barnea M, Wainstein J, Froy O. High caloric intake at breakfast vs. dinner differentially influences weight loss of overweight and obese women. Obesity. 2013;21(12):2504–12. 10.1002/oby.20460 . 23512957

[17] Morgan LM, Shi JW, Hampton SM, Frost G. Effect of meal timing and glycaemic index on glucose control and insulin secretion in healthy volunteers. The British journal of nutrition. 2012;108(7):1286–91. 10.1017/S0007114511006507 . 22176632

[18] Jenkins DJ, Ocana A, Jenkins AL, Wolever TM, Vuksan V, Katzman L, et al Metabolic advantages of spreading the nutrient load: effects of increased meal frequency in non-insulin-dependent diabetes. The American journal of clinical nutrition. 1992;55(2):461–7. . 1734685 10.1093/ajcn/55.2.461

[19] Bertelsen J, Christiansen C, Thomsen C, Poulsen PL, Vestergaard S, Steinov A, et al Effect of meal frequency on blood glucose, insulin, and free fatty acids in NIDDM subjects. Diabetes care. 1993;16(1):4–7. . 8422824 10.2337/diacare.16.1.4

[20] Kahleova H, Belinova L, Malinska H, Oliyarnyk O, Trnovska J, Skop V, et al Eating two larger meals a day (breakfast and lunch) is more effective than six smaller meals in a reduced-energy regimen for patients with type 2 diabetes: a randomised crossover study. Diabetologia. 2014;57(8):1552–60. 10.1007/s00125-014-3253-5 24838678 PMC4079942

[21] Centers for Disease Control and Prevention, National Center for Health Statistics NHANES 2009–2010 [Internet]. Available: http://www.cdc.gov/nchs/nhanes.htm.

[22] Ridker PM. Clinical application of C-reactive protein for cardiovascular disease detection and prevention. Circulation. 2003;107(3):363–9. . 12551853 10.1161/01.cir.0000053730.47739.3c

[23] Meier-Ewert HK, Ridker PM, Rifai N, Price N, Dinges DF, Mullington JM. Absence of diurnal variation of C-reactive protein concentrations in healthy human subjects. Clinical chemistry. 2001;47(3):426–30. Epub 2001/03/10. . 11238292

[24] Matthews DR, Hosker JP, Rudenski AS, Naylor BA, Treacher DF, Turner RC. Homeostasis model assessment: insulin resistance and beta-cell function from fasting plasma glucose and insulin concentrations in man. Diabetologia. 1985;28(7):412–9. . 3899825 10.1007/BF00280883

[25] Bull FC, Maslin TS, Armstrong T. Global physical activity questionnaire (GPAQ): nine country reliability and validity study. Journal of physical activity & health. 2009;6(6):790–804. . 20101923 10.1123/jpah.6.6.790

[26] Almoosawi S, Winter J, Prynne CJ, Hardy R, Stephen AM. Daily profiles of energy and nutrient intakes: are eating profiles changing over time? European journal of clinical nutrition. 2012;66(6):678–86. 10.1038/ejcn.2011.210 22190135 PMC3389619

[27] Sofer S, Stark AH, Madar Z. Nutrition targeting by food timing: time-related dietary approaches to combat obesity and metabolic syndrome. Advances in nutrition. 2015;6(2):214–23. 10.3945/an.114.007518 . 25770260 PMC4352180

[28] Pearson TA, Mensah GA, Alexander RW, Anderson JL, Cannon RO 3rd, Criqui M, et al Markers of inflammation and cardiovascular disease: application to clinical and public health practice: A statement for healthcare professionals from the Centers for Disease Control and Prevention and the American Heart Association. Circulation. 2003;107(3):499–511. . 12551878 10.1161/01.cir.0000052939.59093.45

[29] Rashidi MR, Mahboob S, Sattarivand R. Effects of nibbling and gorging on lipid profiles, blood glucose and insulin levels in healthy subjects. Saudi medical journal. 2003;24(9):945–8. . 12973474

[30] Carlson O, Martin B, Stote KS, Golden E, Maudsley S, Najjar SS, et al Impact of reduced meal frequency without caloric restriction on glucose regulation in healthy, normal-weight middle-aged men and women. Metabolism: clinical and experimental. 2007;56(12):1729–34. 10.1016/j.metabol.2007.07.018. 17998028 PMC2121099

[31] Arble DM, Bass J, Laposky AD, Vitaterna MH, Turek FW. Circadian timing of food intake contributes to weight gain. Obesity. 2009;17(11):2100–2. 10.1038/oby.2009.264 19730426 PMC3499064

[32] Vollmers C, Gill S, DiTacchio L, Pulivarthy SR, Le HD, Panda S. Time of feeding and the intrinsic circadian clock drive rhythms in hepatic gene expression. Proceedings of the National Academy of Sciences of the United States of America. 2009;106(50):21453–8. 10.1073/pnas.0909591106 19940241 PMC2795502

[33] Hughes ME, DiTacchio L, Hayes KR, Vollmers C, Pulivarthy S, Baggs JE, et al Harmonics of circadian gene transcription in mammals. PLoS genetics. 2009;5(4):e1000442 10.1371/journal.pgen.1000442 19343201 PMC2654964

[34] Narasimamurthy R, Hatori M, Nayak SK, Liu F, Panda S, Verma IM. Circadian clock protein cryptochrome regulates the expression of proinflammatory cytokines. Proceedings of the National Academy of Sciences of the United States of America. 2012;109(31):12662–7. 10.1073/pnas.1209965109 22778400 PMC3411996

[35] Wright KP Jr, Drake AL, Frey DJ, Fleshner M, Desouza CA, Gronfier C, et al Influence of sleep deprivation and circadian misalignment on cortisol, inflammatory markers, and cytokine balance. Brain, behavior, and immunity. 2015 10.1016/j.bbi.2015.01.004 . 25640603 PMC5401766

[36] Willet W. Nutritional Epidemiology. Ney York, NY: Oxford University Press; 1998.

[37] Ockene IS, Matthews CE, Rifai N, Ridker PM, Reed G, Stanek E. Variability and classification accuracy of serial high-sensitivity C-reactive protein measurements in healthy adults. Clinical chemistry. 2001;47(3):444–50. . 11238295

